# Management of Somatization in COVID-19: A Narrative Review

**DOI:** 10.7759/cureus.30262

**Published:** 2022-10-13

**Authors:** Dhanashree Wankhade, Ashish P Anjankar

**Affiliations:** 1 Medicine, Jawaharlal Nehru Medical College, Datta Meghe Institute of Medical Sciences, Wardha, IND; 2 Biochemistry, Jawaharlal Nehru Medical College, Datta Meghe Institute of Medical Sciences, Wardha, IND

**Keywords:** antidepressants, depression, anxiety, covid-19 pandemic, distress, somatization

## Abstract

Somatization refers to the condition in which psychological distress is shown in the form of somatic symptoms such as persistent headache, nausea, gastrointestinal discomfort, etc. Various predisposing factors, including familial such as high expressed emotion, poor parental care, genetic, biological, and demographic which includes age and gender, cognitive such as learning disabilities, psychiatric such as depression, anxiety, post-traumatic stress disorder, social, etc., play an essential role in saturation of the disease. During the time of the COVID-19 pandemic, psychological distress increased in the patients infected with the coronavirus due to some the factors such as social distancing from loved ones, lack of physical exercise, loss of income, loneliness due to quarantine, etc. Therefore, management and treatment of the disorder became essential, especially in coronavirus-infected patients, as it may lead to an increase in complications of the disease. Many studies have been conducted to identify the proper way to manage the condition. Treatments include pharmacological therapy and psychosocial interventions. Pharmacological therapy includes using various antidepressants, hypnotics, and sedatives such as benzodiazepines. For the treatment, mirtazapine is a secure and reliable antidepressant. Another drug, trizolobenzodiazepine adinazolam, was also very useful in treating patients. In some randomized experiments, alprazolam significantly outperformed amitryptiline in reducing the symptoms. Psychosocial interventions include sessions such as cognitive behavioral therapy (CBT), mindfulness-based cognitive therapy, relaxation training, meditation, and psychological interventions such as enhancing multidimensional social help, modifying cognitive assessment, directing positive coping, and inspiring positive emotions.

## Introduction and background

When psychological distress is expressed in the form of physical symptoms, it is referred to as somatization [1]. The diagnosis of somatization is problematic because it can be confused with other systemic diseases; therefore, patients with somatization tend to move from one medical practitioner to another for the correct diagnosis. For early diagnosis of the disease, history should be taken carefully [1]. The doctor should manage the patient with proper treatment and care and deal with the patient by setting reasonable treatment goals empathetically. For the patient with a late diagnosis, the doctor should set the limit and treat accordingly [1]. Patients and doctors usually become frustrated when the diagnosis is not reached, and the complaints continue as it is and still persist after assessment and treatment [2]. About 15-20 percent of the time, explanations for medical complaints such as fatigue, malaise, dizziness, and abdominal discomfort can be found. About 20 percent of the physical symptoms cannot be explained and are therefore called unexplained physical symptoms [2]. \

The aetiology of somatization is from both psychology and medicine. In the classification of somatoform disorders, according to the diagnostic and statistical manual of mental disorders (DSM), there are conditions such as somatization disorder, undifferentiated somatoform disorder, conversion disorder, hypochondriasis, and pain disorder, body dysmorphic disorder, somatoform disorder not otherwise specified [2]. The patients of somatization do not produce the symptoms intentionally; they somatize unconsciously as a dysfunctional coping mechanism [2].

The ability of a person to respond to extreme stress is known as psychological resilience. In the studies conducted in China, the findings point to a significant frequency of psychological discomfort in the general community at the height of COVID-19 [3]. Indicators of distress, anxiety and somatization had moderately increased levels after experiencing clinical COVID-19 symptoms, indicating a serious illness that has been experienced. The existence of chronic fatigue syndrome brought on by the preceding COVID-19 disease is likely what is responsible for the significantly higher level of clinical depression in our study. The study's findings demonstrated that young patients with COVID-19, even with a modest and low-symptom course, may experience side effects such as discomfort, anxiety, somatization, and other issues. Further research is needed to determine the causes, timing, and potential risk factors for their development [4].

## Review

During the pandemic, various factors, including social distancing, were responsible for obsessive-compulsive traces and somatization. As a result, there are statistically widespread risks for psychic tension, emotional disturbances, and sleep disturbances in the battle against COVID-19; however, the exact cause of these disorders has not yet been determined [5]. Therefore, better-designed cohort studies or randomized clinical trials are required to investigate their most significant linkages [5]. The physical symptoms of somatization are very inconsistent with history, laboratory findings, physical examination and other investigations [6]. After the severe acute respiratory syndrome (SARS) outbreak, the COVID-19 epidemic is the most significant outbreak. Hence, an extensive range of psychological suffering during the outbreak was indicated by prior studies [7]. Chronic disease history is only a factor that causes mild somatization [7]. At the same time, some patients experienced somatization and panic attacks as a result of their history of chronic diseases, male gender, and related demographic characteristics [7]. Unexpectedly, comorbid somatization was more common in men than women [7]. Socio-economic factors as well as demographic factors, such as gender, marital status, employment state, illness etc., comprise the risk factors for the disease [8]. Anxiety may cause visceral allergies, which result in gastrointestinal tract discomfort [8]. Along with somatization, insomnia was also noticed in patients with COVID-19 [8]. Somatization is associated with a history of trauma and post-traumatic stress disorder [9]. Other predisposing factors during the pandemic were changes in lifestyle, isolation, social distancing, reduction in physical activities, sleep impairment, and poor diet. This is shown in (Table 1) [9].

**Table 1:** Predisposing factors of somatization with examples The above-mentioned factors contribute to the precipitation of somatization [9].

Type of predisposing factor	Examples
Biological and genetic	Sensitive sympathetic nervous system, Overactive hypothalamo-pituitary axis
Familial	Poor parental care and modelling, High expressed emotion, Parent-child conflict
Social	Difficulty with interpersonal relationship
Psychiatric	Depression, Anxiety, Post-traumatic stress disorder
Demographic	Gender, Age
Cognitive	Learning disabilities, Low intelligence

Cognitive Behavioural Therapy (CBT)

A notable finding was that all psychopathological symptoms were linked to loneliness, possibly as a result of isolation and quarantine regulations and a lack of social support [10]. Furthermore, those who are isolated frequently find it unpleasant as they are cut off from their loved ones, lose their liberties, and lose interest at home, these factors could all make a contribution to loneliness [10]. In guidelines for mental fitness, there is a need to define the essential period of individual isolation, communicate the motives for isolation in detail, provide the isolated people with sufficient substances which are needful, and during the duration of isolation, offer intellectual fitness interventions to the impacted patients [10]. Building resilience through cognitive behavioural therapy can help combat anxiety, sadness, somatization, and incapacitating symptoms, as well as the social isolation that has been a hallmark of COVID-19 and comparable pandemic disorders [11]. The results imply that cognitive behavioural therapy can be useful for people with somatization disorder and should be the topic of a carefully monitored therapy trial [12]. A portion of the general population may experience health problems as a result of loss of income or social isolation [13]. The prevalence of psychological issues was tightly correlated with the patient's personal characteristics, environment, and social network. For COVID-19 patients, it is crucial to deliver timely psychological care services and keep track of them for a more extended time [14].

Contrary to the findings of various studies that only describe the presence of a notable amount of negative emotions during the pandemic, the presence of positive emotions like assurance, calmness, relaxation, and happiness was not seen; on the other hand, negative emotions were seen to arise either suddenly or gradually. Most of our participants thought that the support provided by patients, families, team members, the government, and social groups altogether was associated with happy emotions [15]. According to studies, positive feelings are crucial for psychological trauma recovery and adjustment [15]. Disaster-related psychological trauma can be avoided, and optimism can help with psychological recovery from post-traumatic stress disorder [15]. It is recommended to enhance comprehensive social support, change cognitive assessment, direct positive coping mechanisms, and develop a positive attitude in order to resist a psychological pandemic [15]. The concern of the disease spreading to other members of the family and their loved ones may also explain the variation in the intensity level of the psychiatric signs amongst married patients. Patients who obtain satisfactory outcomes from nucleic acid checks also experience more significant clinical depression. In patients who needed oxygen inhalation, somatization symptoms worsened [16].

Psychological suffering is amplified when a family member or acquaintance passes away as a direct result of SARS-CoV-2 illness [17]. People with a prior history of mental illnesses appear to be significantly more susceptible to this effect [17]. To reduce the effects of the pandemic, mitigation efforts should focus on the population that has suffered losses [17]. Once the acute infection has been treated, immediate psychological support and intervention are required. The COVID-19 outbreak may cause psychological issues in both healthcare providers and non-providers, which could manifest as physical symptoms [18]. Patients on the transplant waiting list experience elevated somatization symptoms related to COVID-19 worries during the first lockdown of the COVID-19 epidemic. They require psychological counselling as a susceptible population to enhance their mental health under stressful situations [19]. In the research on anxiety disorders and major depressive disorder, the concept of intolerance of uncertainty (IU) is becoming more prevalent [20].

Pharmacotherapy

Mirtazapine positively impacts the patients of somatization [21]. In pretreatment assessments, 41.3 percent of the sample gave this explanation, but by three months after treatment, this number had significantly increased to 63.9 percent. At the beginning of the study, nearly half of the sample (48.52 percent) was taking benzodiazepines, but only 6.71 percent of the patients still required them three months later. About 13.36 percent of the patients experienced negative consequences. 4 percent of all dropouts were attributable to unfavourable circumstances. For the treatment of depression accompanied by somatic symptoms, mirtazapine is a reliable and secure antidepressant with the ability to change the way somatizing patients attribute their somatic symptoms [21]. There was some evidence suggesting the efficacy of the various antidepressant classes was similar, but it was scarce and of poor to low quality [22]. The impact of pharmaceutical therapies on somatization in patients having severe psychological conditions such as a major type of clinical depression is examined in two types of investigations. The first was a major depressive episode patient-controlled, double-blind experiment [23]. Trizolobenzodiazepine adinazolam was used to treat their clinical depression. Patients reported a vast improvement in a somatization or tension rating in addition to a considerable improvement in their depression. The consequences of a double-blind, randomized experiment contrasting amitriptyline with alprazolam in patients with predominant depressive episodes have been mentioned in the second examination. Alprazolam appreciably outperformed amitriptyline in this study’s six-week somatization experiment [23].

The most effective treatment for a number of somatoform disorders is cognitive behavioural therapy (CBT), while studies have also shown some benefits of consulting a primary care provider [24]. To encourage good mental health outcomes and emotional tolerance in all communities, access to mental health services and supports should be widened. Finding those who are most vulnerable and getting them the care they need is essential. Recognizing COVID-19's psychopathological variances. Assessing the COVID-19's temporal effects on mental health [25]. The development of methodologies and measurements. Encouraging multidisciplinary research in mental health. Putting an emphasis on mental health research and evidence mapping. Creating strategies and interventions for mental health. Putting mental health first and incorporating it into current healthcare systems [25]. Enhancing accessibility to information and resources for mental health. Mobilizing organizations and social services in the neighbourhood. Strengthening mechanisms for mental health in preparation for COVID-19 and other public health crises [25].

Identifying elements linked to successful therapeutic outcomes is helpful for creating effective treatment plans [26]. The resident used the following hospital rating system to analyse patients' responses to treatment at the time of discharge from the hospital; if there is no improvement, some improvement, or remarkable improvement, which means the resolution of symptoms and satisfaction at social and occupational levels [26]. Paediatricians, for example, can instruct parents and young people to enhance physical activity in order to boost their emotional and physical well-being [27]. These experts should also examine if a child is engaging in an acceptable amount of physical exercise in clinical settings. To avoid somatization in kids and teens, it is crucial to provide accurate information, prevention advice, and mental health referrals [27]. Patients suffering from somatization may experience some level of joint pain. The patient's perception of pain should guide symptomatic relief therapy, not radiographic findings. The primary subjective experience of pain may change between two patients with comparable joint illness who report different levels of pain, or it may be the same primary experience but expressed differently [28].

Single-session treatment can be very successful in lowering anxiety when provided in the context of a medical visit by paediatric healthcare professionals [29]. Given the close relationship between one's actions and feelings, cognitive-behavioural therapy promotes behaviour modification to reduce anxiety and depression [29]. Additionally, short therapy is most beneficial when used in conjunction with a specific stressor, such as COVID-19 [29]. Before starting the treatment and management of the condition, we first clarify the presenting problems and their pathology and psychopathology. To review the patient, a diagnosis of DSM-IV SD is made by the clinician. The implementation of affective cognitive behavioural therapy (ACBT) caused significant improvement in the patients. The benefits of this method are maintained for at least one year after the treatment has ended. The components of affective cognitive behavioural therapy covers behavioural management, relaxation training, emotion identification and regulation, cognitive reorienting, and interpersonal skill training [30]. Investigations of the function of emotional avoidance as a mediator of the therapeutic effects of somatization have been studied. Both treatments that are cognitive behavioural therapy and dialectical behavioural therapy were successful; however, those who underwent dialectical behavioural therapy experienced longer-lasting reductions in emotional avoidance than those who received cognitive behavioural therapy [31].

The increase in psychosomatic symptoms during the COVID-19 outbreak was found to be influenced by changes in biological rhythm and perceived threat [32]. The various body-mind relationship disturbance were recently grouped into various interventions, suggesting that in some cases, the stress associated with uncertainty worsened a number of biological disorders with distinct pathophysiology. While in other cases, increased sensitivity to physical stimuli combined with hyper-reactivity of the autonomic system forms a cycle of learning processes involving biological and psychological factors [32]. Setting up resources and support systems for anyone who might need them is crucial. We can learn from previous instances of a such nature, like the SARS pandemic [33]. Future studies should examine how this symptomatology changes through the years and whether it is sustained or resolved [33]. A clinical strategy that takes into account the patient's overall life context, strategies which include naming the illness using language that is mutually expressive, normalizes the patient's physical discomfort, applies a chronic disease model, which is a model with key elements of a health care system that encourage high-quality chronic disease care and its proper functioning, fulfils the physician's need for efficacy and certainty. By appropriately limiting access to medical treatments, health systems can facilitate care coordination and reduce iatrogenic harm [34].

The management of somatization is not easy as the patient is already distressed; therefore, there are various steps in managing the patient with somatoform disorders; these include the following steps. Firstly we need to understand the patient and make them feel understood, then change the agenda if required and lastly, make the link with the patient [35]. The treatment includes cognitive strategies like controlled breathing and guided imagery. Other methods include various relaxation techniques [35]. Mindfulness-based cognitive therapy is also helpful in treating these patients. The first step in this approach is to become aware of any unpleasant sensations or emotions and start the treatment accordingly. This process can lead to the alteration of harmful behavioural habits and an improvement in self-care and self-compassion [36]. The medical leadership of the training programme must adopt a biopsychosocial paradigm; it should be demonstrated through role modelling and included in the curriculum. The curriculum hierarchy of somatization in primary care. This is shown in (Table 2) [37].

**Table 2:** The curriculum hierarchy of somatization in primary care The table gives an idea about various curriculum issues with its markers [37].

Curriculum issue	Curriculum markers examples
Integrated care model	Multidisciplinary faculty
The control of countertransference	Case supervision
Skills of interviewing and managing the patient	Somatization role play
Psychiatric skills and defining terms	Somatization seminars
System review of psychosocial factors	An objective review of resident interviews
Biopsychosocial model	Written curriculum

An intensive treatment plan that also included individual and group cognitive behavioural therapy, relaxation training, and other medical and psychological interventions. It also included interventions which are specific to symptoms, such as management of depression in group therapy or psychology-related pharmacotherapy, if indicated [38].

## Conclusions

In the emerging context of COVID-19, psychiatric distress in patients of somatization was more triggered during the COVID-19 pandemic due to certain factors like social distancing, lack of physical activity, socioeconomic situations such as loss of income, etc.; therefore, management of this disorder in patients is crucial. Psychological counseling of susceptible populations should be done to enhance mental health under the stressful situation of the COVID-19 pandemic. Management of somatization includes pharmacological therapy like the use of hypnotics, sedatives, and antidepressant drugs. Benzodiazepines were proven to be effective. Alprazolam also showed promising results. Psychosocial interventions such as cognitive behavioral therapy (CBT), and mindfulness-based cognitive therapy proved helpful. In addition, relaxation training, meditation, and inspiring positive emotions should be recommended to manage the disorder during the period of the COVID-19 pandemic.
